# mHealth technologies in research studying cardiovascular health in cancer: A systematic review

**DOI:** 10.1371/journal.pdig.0001027

**Published:** 2025-09-25

**Authors:** Roberto M. Benzo, Anvitha Gogineni, Macy K. Tetrick, Rujul Singh, Peter Washington, Soledad Fernandez, Electra D. Paskett, Frank J. Penedo, Sanam Ghazi, Alex Osei, Steven K. Clinton, Jessica Krok-Schoen, Sarah Weyrauch, Daniel Addison

**Affiliations:** 1 Division of Cancer Prevention and Control, Department of Internal Medicine, College of Medicine, The Ohio State University, Columbus, Ohio, United States of America; 2 The Ohio State University Comprehensive Cancer Center, The Ohio State University Wexner Medical Center, Columbus, Ohio, United States of America; 3 Division of Clinical Informatics and Digital Transformation, Department of Medicine, University of California San Francisco, San Francisco, California, United States of America; 4 Department of Biomedical Informatics and Center for Biostatistics, Ohio State University, Columbus, Ohio, United States of America; 5 Departments of Psychology and Medicine, University of Miami, Coral Gables, Florida, United States of America; 6 Sylvester Comprehensive Cancer Center, Miller School of Medicine, University of Miami, Miami, Florida, United States of America; 7 Cardio-Oncology Program, Division of Cardiology, The Ohio State University Medical Center, Columbus, Ohio, United States of America; 8 Department of Design, The Ohio State University, Columbus, Ohio, United States of America; 9 Division of Medical Oncology, Department of Internal Medicine, The Ohio State University, Columbus, Ohio, United States of America; 10 School of Health and Rehabilitation Sciences, College of Medicine, The Ohio State University, Columbus, Ohio, United States of America; 11 Division of Epidemiology, College of Public Health, Ohio State University, Columbus, Ohio, United States of America; Iran University of Medical Sciences, IRAN, ISLAMIC REPUBLIC OF

## Abstract

Cancer survivors face an increased risk of cardiovascular disease (CVD) due to treatment-related toxicity, lifestyle factors, and comorbidities. Addressing CV health is crucial for improving quality of life and long-term outcomes. The American Heart Association’s Life’s Essential 8 framework highlights modifiable determinants of CV health, emphasizing early detection and monitoring. Mobile health (mHealth) technologies, such as wearables and smartphone apps, offer continuous tracking, yet their applications in cancer survivorship remain unclear. This review systematically characterizes the types of mHealth technologies used to monitor CV health in cancer survivors, focusing on the specific data collected (major adverse CV events, CV risk factors, and surrogate endpoints) and the use of active versus passive collection methods. A systematic search of PubMed, Scopus, Embase, and Web of Science identified studies published between January 1, 2016, and June 13, 2024. Eligible studies included observational and interventional designs assessing at least one CV outcome using mHealth. Data were extracted on design, technology type, and outcomes. Risk of bias was evaluated using the Cochrane RoB-2 and ROBINS-I tools. Fourteen studies (13 interventional, one observational) met criteria. Physical activity was the most monitored risk factor, followed by HR. The most common technologies were mobile apps and commercial wearables. Passive methods typically captured PA and HR, while active methods captured PA, symptom tracking, and diet. A key finding was the lack of integration with electronic medical records, highlighting a gap in clinical implementation. mHealth provides scalable tools to track CV health indicators in cancer survivors. Findings highlight the potential to support practice by enabling remote oversight of risk-reducing behaviors and guiding lifestyle interventions. However, we also identified gaps, including the underutilization of biomarkers (e.g., HRV) and the lack of integration with electronic records. Future research must address these gaps to translate real-time data into clinical insights and optimize survivorship care.

## Author summary

Cancer survivors are at a significantly higher risk of developing heart disease, partly due to the effects of cancer treatments on the heart and common risk factors such as physical inactivity, poor diet, and high blood pressure. Monitoring heart health early is crucial for improving quality of life and preventing serious complications. Mobile health (mHealth) technologies—such as wearable fitness trackers and smartphone apps—offer a promising way to track key health indicators, including physical activity, heart rate, and symptoms in real time. This systematic review explores how mHealth technologies are being used to monitor heart health in cancer survivors. We identify the types of data these tools collect and evaluate their role in early detection of cardiovascular issues. While mHealth has great potential to enhance survivorship care, challenges remain in integrating these technologies into routine clinical practice. Our findings provide insights for researchers and healthcare professionals on leveraging digital health tools to improve cardiovascular health in cancer survivors.

## Introduction

Cardiovascular disease (CVD) is the leading non-malignant cause of death among cancer survivors [1]. Recent evidence shows that cancer survivors face a greater risk of cardiovascular (CV) mortality than the general population [2], with studies showing a 37% higher risk of incident CVD and a 52% higher risk of heart failure compared to individuals without a cancer diagnosis [2–5]. This increased risk is influenced by cancer type, treatment-related toxicity, health behaviors (e.g., physical inactivity, smoking), and existing comorbidities (e.g., metabolic syndrome). The American Heart Association’s (AHAs) “Life’s Essential 8 (“LE8”)” framework highlights several modifiable determinants of CV health, including smoking, obesity, physical inactivity, poor diet, inadequate sleep, unhealthy blood pressure (BP) range, cholesterol, and blood glucose [6–8]. Recognizing and managing cardiotoxicity from across the cancer continuum (diagnosis, treatment, remission) is complex due to these varied risk factors, which can lead to severe complications. Given this heightened vulnerability, current clinical monitoring strategies are often insufficient, challenging for both patients and providers to implement, and frequently overlook the subtle, developing signs of CVD. To address these critical gaps and facilitate timely interventions for improved health-related quality of life (HRQoL), innovative approaches are urgently needed.

Mobile health (mHealth) technologies, including wearable devices and smartphone applications, have emerged as promising tools that allow continuous, real-time monitoring of health outcomes [9–12]. First, they enable continuous monitoring between clinic visits, which is very important given that cardiotoxicity can develop months after treatment completion and may initially present with subtle, subclinical changes [13]. The remote and continuous data collection enabled by wearable devices, in particular, can detect early physiological alterations, such as changes in heart rate variability (HRV), activity patterns, or BP trends, that may precede symptomatic CV events, potentially allowing for earlier intervention [14]. Furthermore, these technologies also improve accessibility to CV monitoring for survivors who may face mobility limitations due to treatment-related fatigue or those living in rural areas with limited access to cardio-oncology services [15]. Finally, mHealth tools can simultaneously capture multiple measures of objective physiological data through sensors and subjective patient-reported outcomes via integrated apps, providing a more comprehensive picture of CV health that encompasses both clinical markers and quality of life measures [16].

Despite their rapid adoption, there is limited information regarding their specific applications for monitoring CV health in cancer survivors, particularly given the recent rise in both cancer treatment-related cardiotoxicities and mHealth advancements. Existing cancer-related mHealth studies vary widely in focus, from tracking physical activity (PA) and heart rate (HR) to monitoring diet and symptoms indicative of CV risk [9,11,12]. This inconsistency and fragmented approach across studies means that a systematic synthesis of mHealth applications used to study CV health in cancer research is lacking, leaving a gap in understanding the types of mHealth technologies employed, their feasibility, the impact of preventative behaviors, and the specific CV-related data they capture.

This systematic review aims to bridge this gap by evaluating how mHealth technologies collect CV-related data in both observational and interventional studies of cancer survivors. We focus on CV outcomes, risk factors, and surrogate endpoints, distinguishing between active and passive data collection methods.

## Methods

This systematic review is reported in accordance with the Preferred Reporting Items for Systematic Reviews and Meta-Analyses statement (2020) (S1 Checklist) [17]; and the Cochrane Handbook for Systematic Reviews of Interventions [18]. The protocol is registered with PROSPERO (CRD420250651090).

### Search strategy

Relevant literature was systematically searched and evaluated from four databases: PubMed, Scopus, Embase, and Web of Science, in adherence with the Preferred Reporting Items for Systematic Reviews and Meta-Analyses (PRISMA) guidelines. The same search query was applied across all databases to maintain consistency and ensure comprehensive coverage. The search terms combined concepts related to CV health, mHealth technology, cancer, and study design. The final query used was: (cardi*) AND (mHealth OR mobile health) AND (cancer OR oncology) AND (cohort OR cross-sectional OR intervention OR randomized controlled trial OR observational OR experimental).

### Eligibility criteria

Studies were screened using predefined inclusion (IC) and exclusion criteria (EC) described in Table 1. Inclusion criteria were: (1) prospective trials published from January 1, 2016, onwards (through 06/13/2024). Our selection of the 2016–2024 time range was guided by converging trends in the maturation of consumer and clinical mHealth technologies post-2016, alongside a sharp rise in cardio‑oncology publications and analyses beginning in 2016 [19,20]; (2) studies involving cancer survivors, which is the focus of this study (3) inclusion of adults in the sample (studies with mixed adult and pediatric populations were included as long as adults were part of the sample), (4) observational or experimental study design, (5) measurement of at least one CV health-related outcome using an mHealth technology, as this criterion directly aligns with our objective to evaluate how mHealth tools collect CV-related data (6) publication in a peer-reviewed journal, and (7) availability of a full-text version in English. We excluded conference abstracts and review articles. mHealth technologies were defined as any health-focused wearable device, online assistance tool, or smartphone application.

**Table 1:** Inclusion and exclusion criteria.

Inclusion criteria	Exclusion criteria
IC1: Publication date: 2016–2024	EC1: No inclusion of CV outcomes, risk factors, or surrogate end points measured actively or passively using mHealth technology
IC2: Cancer survivors or have cancer currently	
IC3: Adults (18+)	
IC4: Intervention, clinical trial, observational, experimental, cross sectional, or cohort study	EC2: Case reports, case series, reviews, editorials, letters, commentaries, or protocol studies
IC5: CV outcomes, risk factors, or surrogate end points measured actively or passively using mobile health technology	EC3: Non-peer-reviewed articles, conference abstracts, or used a pre-existing dataset
IC6: Peer reviewed, full text	
IC7: English language	

**Abbreviations:** CV, cardiovascular; IC, inclusion criteria; EC, exclusion criteria; mHealth, mobile health.

The CV health-related outcomes in this study were established a priori and included three categories (a) major adverse CV events (MACE), (b) risk factors, and (c) surrogate endpoints. The MACE included arrhythmias, sudden death from CV disease, hospitalization from CV disease, or coronary revascularization. The risk factors reflected those described by the American Heart Association (AHA)’s Life Essential 8 [6,7], which include diet, PA, tobacco, sleep, weight, body composition, cholesterol, blood glucose, and BP [21]. Surrogate endpoints included HR, cardiorespiratory fitness (CRF), signs or symptoms potentially indicative of CV health or risk (e.g., weight change, shortness of breath, syncope), and metabolic syndrome.

To categorize the mHealth technologies included in this review, technologies were grouped into seven distinct categories: apps and online platforms, commercial wearables (e.g., fitness trackers, smartwatches, wearable continuous glucose monitors), other commercial devices (e.g., BP cuffs, smart speakers, tablets, and digital weight scales), research-grade trackers (e.g., ActiGraph and activPAL), electronic medical records (EMRs), online surveys, and mobile phones (i.e., supporting telephone calls and text messages). This categorization highlights the breadth of technologies employed to measure survivors’ CV health, integrating consumer-focused tools and research-oriented devices. These devices enabled the collection of both active and passive data. Active data are typically obtained when participants actively provide information about their health, behavior, or other relevant details through mHealth technology [22]. In contrast, passive data comprises sensor-generated and system-usage data collected automatically by mHealth technology, often with minimal (e.g., wearing a device) to no user input [22].

Devices such as Bluetooth-enabled BP cuffs and weight scales were classified as passive mHealth technologies because the data from these devices are typically collected and transmitted automatically to a connected device or platform without requiring the user to input the information manually. The user’s primary action is limited to stepping on the scale, with the actual data transmission occurring passively in the background [23].

Other commercial devices are defined as consumer-grade devices designed for health monitoring that do not require direct attachment to the body during use. These devices include but are not limited to commercially available BP monitors, glucose monitors (non-wearable), tablets, and other tools designed for health-related data collection or feedback. Unlike wearable devices, which are continuously worn on the body (e.g., fitness trackers, smartwatches), non-wearables are used intermittently and typically involve standalone usage or interaction with the user at specific points in time.

Online surveys were regarded as mHealth technologies when incorporated into mobile platforms or applications, enabling real-time data collection, symptom tracking, or support for behavioral interventions. However, standalone online surveys that did not report mobile optimization or integration with other mHealth tools were excluded from this classification. Surveys completed during in-person visits were not considered to utilize mHealth technology.

Telephone calls were classified as mHealth technologies when used for health-related purposes such as remote monitoring, counseling, or intervention delivery via mobile or wireless networks. In contrast, traditional landline calls without a mobile or digital component did not meet the criteria for mHealth. Given that all included studies were published within recent years, we assumed the use of a mobile device when the article did not specify whether telephone calls were made via a landline or a mobile phone.

### Screening and selection process

We conducted a thorough review of studies using Covidence Systematic Review Software (Vertias Health Innovation, Melbourne, Australia, www.covidence.org) [24], structured in three distinct phases. In the first phase, we uploaded all identified studies and removed duplicates. The second phase involved screening the titles and abstracts of each study to apply our predetermined inclusion and exclusion criteria. In the final phase, we performed an in-depth review of the full texts of the remaining studies. At least two reviewers (RB and AG) filtered both the abstract and title screenings and conducted the full-text review. All reviewers agreed on the inclusion and exclusion criteria for screening the studies. Disagreements between the two reviewers during the title/abstract and full-text screening were first discussed to identify the source of disagreement (e.g., interpretation of inclusion/exclusion criteria). If the reviewers could not resolve the disagreement, other reviewers were consulted (MT, SG). The predefined inclusion and exclusion criteria guided the resolution developed a priori. When criteria required additional judgment (e.g., outcome specificity), decisions were based on whether the study aligned with the research question and objectives of the review. All decisions were documented for transparency and reproducibility.

### Data extraction

Data extraction was systematically conducted by two reviewers (RB and AG) using a standardized, pre-piloted form, with discrepancies resolved by a third reviewer (MT). The focus of data extraction was to gather comprehensive information relevant to our primary objective of characterizing mHealth interventions for CVD prevention and management. The following variables were extracted: (1) Study characteristics, which included publication year, purpose, study design (e.g., randomized controlled trial, quasi-experimental), sample size, participant cancer site, and study duration. These details provided insights into the context and generalizability of the findings, as well as for assessing potential sources of bias; (2) types of CV measures, such as major adverse CV events (MACE), CV risk factors (e.g., PA, weight, BP), and surrogate endpoints (e.g., HR, signs/symptoms). These specific measures were prioritized as they directly align with established CV risk factors and clinical endpoints, enabling a thorough evaluation of the application of mHealth interventions on CVD prevention and management; (3) types (passive and active) of mHealth technologies used (e.g., smartphone applications, telemedicine platforms, wearable sensors) along with their key purpose (e.g., monitoring, communication, education). This allowed us to characterize the diverse landscape of mHealth interventions and identify prevalent technological approaches in CVD care.

### Risk of bias in individual studies

Studies assessed for bias were led by the two reviewers (RB and AG) using the Cochrane Risk of Bias tool for randomized trials (RoB-2) for randomized controlled trials [25], and the Risk of Bias in Non-randomized Studies of Interventions (ROBINS-I) tool for non-randomized or observational studies [26]. Figures illustrating the summary of bias are shown in the manuscript and were created using the Risk-of-bias Visualization (robvis) tool [27]. For studies assessed with the RoB-2, each of the five domains (bias arising from the randomization process, bias due to deviations from intended interventions, bias due to missing outcome data, bias in measurement of the outcome, and bias in selection of reported results) was critically appraised. Specifically, for blinding, we assessed whether participants and personnel were aware of the assigned intervention and whether outcome assessors were blinded to the intervention groups, acknowledging that blinding participants and personnel can be particularly challenging in mHealth interventions due to the nature of technology use. For studies assessed with ROBINS-I, bias was evaluated across seven domains (i.e., confounding, classification of interventions, selection of participants into the study, deviations from intended interventions, missing data, measurement of outcomes, and selection of the reported result). Challenges encountered included the heterogeneity of mHealth interventions, which sometimes made the standardized application of criteria more difficult.

### Analysis

No data were pulled from studies to conduct additional statistical analyses. This review focused on summarizing the prevalence of studies that met the inclusion/exclusion criteria, and their characteristics related to using mHealth to measure CV outcomes.

## Results

This section presents the findings of our systematic review, organized to reflect the logical progression from study identification to specific outcomes. We first detail the study selection process, followed by characteristics of the included studies, their quality assessment, and finally, the types of CV measures collected using mHealth technologies.

### Study selection

**Fig 1:**
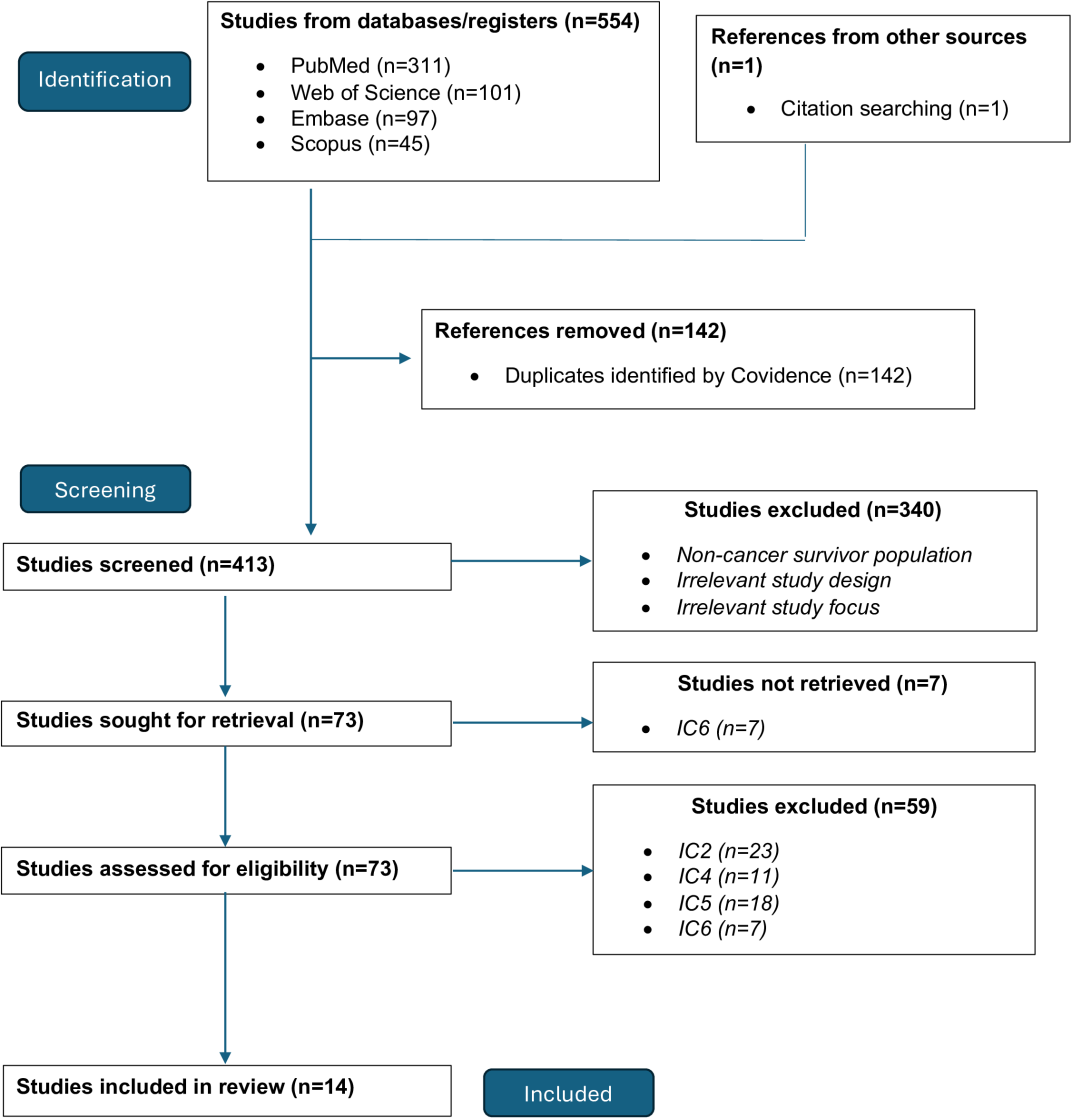
PRISMA flow diagram.

The PRISMA flow diagram outlining our study selection process is shown in Fig 1. From an initial database search, 554 studies were identified, and one additional study was found through citation searching. Following the removal of 142 duplicate references, we commenced the screening process with 413 unique studies. During the “initial screening” phase, our team reviewed only the abstracts to assess relevance based on three core criteria: 1) focus on CV health, 2) utilization of digital health technologies, and 3) a study population consisting of cancer survivors. This initial screening narrowed our selection to 73 studies. Of these 73 studies, 23 studies were excluded for not including a cancer population (IC2), 18 were excluded for failing to investigate a CV-related measure actively or passively via mHealth (IC5), 11 were excluded for not conducting an observational or experimental study (IC4), and seven were excluded because the full-text version of the study could not be retrieved (IC6). Ultimately, 14 studies met the inclusion criteria for analysis, covering various mHealth technologies used to assess CV health in cancer populations.

### Study characteristics

Table 2 summarizes the number of studies identified, screened, included by the database, and total. PubMed contributed the largest number of unique studies (N = 311). After screening titles and abstracts, 53 studies remained eligible for further review, with 10 ultimately meeting the full-text inclusion criteria. One of the excluded studies from the PubMed database was a protocol (EC2), which led us to its most recent publication that met our eligibility criteria [28]; this was also noted as citation searching in the PRISMA (*see* Fig 1)*.* Scopus initially yielded 45 studies, which were reduced to 23 after removing duplicates; four proceeded to full screening, and one met the inclusion criteria. Embase identified 97 studies, narrowing to 43 unique records after duplicates were removed; 10 proceeded to full screening, and two were included in the final review. Web of Science initially retrieved 101 studies, resulting in 35 unique records after duplicate removal; six were further reviewed, and one was included. Overall, PubMed contributed the most studies (N = 10) in the final selection. Of the 14 studies that met the inclusion criteria, 13 were interventional [23,28–39], and one was observational [40].

**Table 2:** Database breakdown.

Database	Initial papers	After duplicates removed	Passed title & abstract screening	Passed full-text review
PubMed	311	*312	53	10 (3.2%)
Scopus	45	23	4	1 (4.3%)
Embase	97	43	10	2 (4.7%)
Web of Science	101	35	6	1 (2.86%)
Total	554	413	73	14 (3.39%)

Note: * One additional study was identified (i.e., citation searching) and included which resulted from the PubMed search.

**Fig 2:**
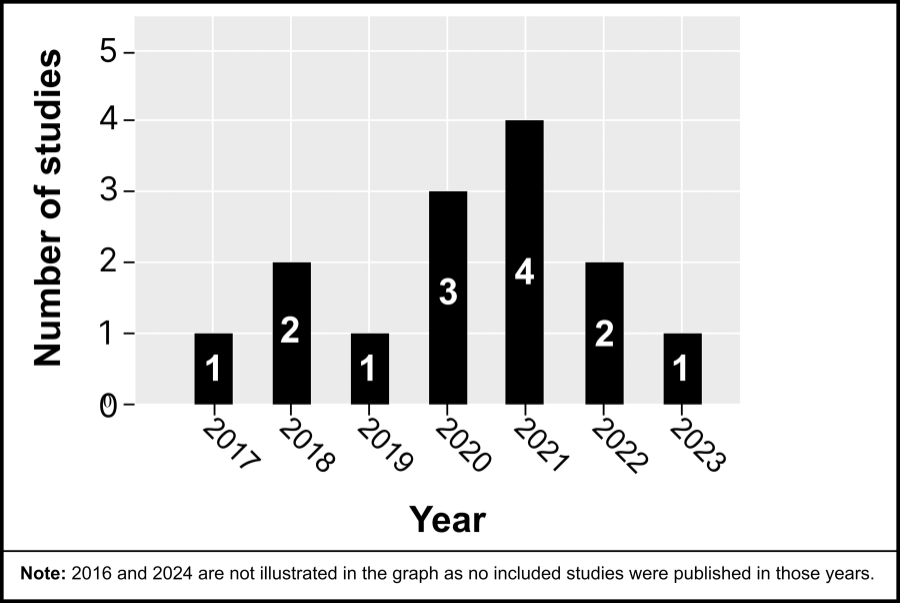
Reviewed studies by year of publication.

The studies included were conducted between 2017 and 2023, with most published in 2021 (*see* Fig 2). Studies consisted of sample sizes ranging from 7 to 356, with 1,194 participants in this review. Six studies were conducted in the USA [23,28,29,31,37,39], three in the Netherlands [32,33,40], three in Korea [34,36,38], one in the United Kingdom [30], and one in Canada [35].

### Participant characteristics

**Fig 3:**
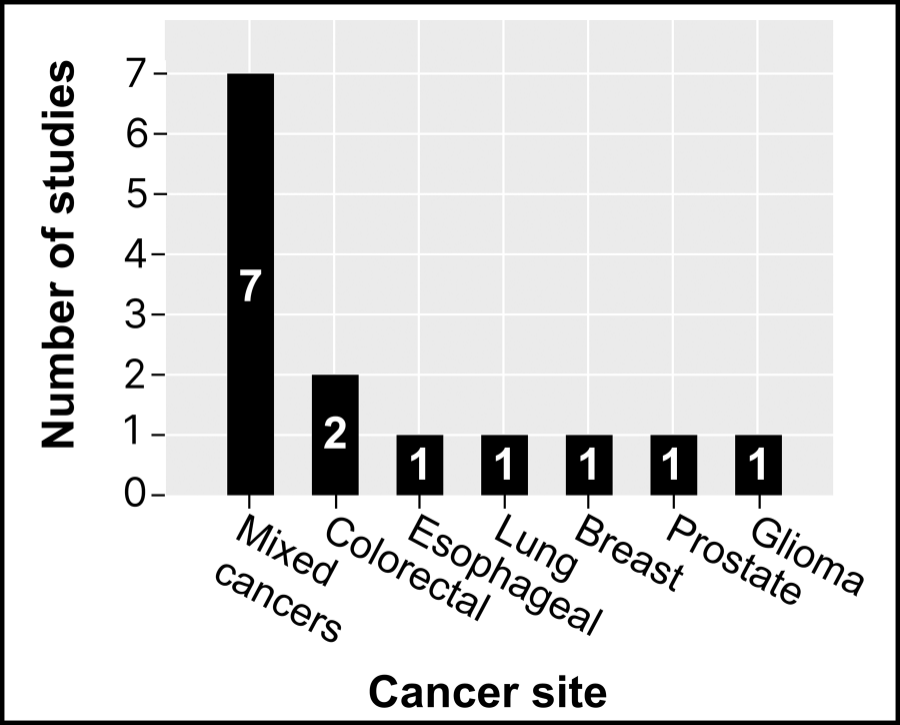
Reviewed studies categorized by cancer site.

Fig 3 illustrates the distribution of participants’ cancer types across the included studies. The majority of studies (n = 7) included participants with a variety of cancer types, representing mixed cancer populations [23,28,29,31,32,39,40]. Two studies specifically focused on individuals with colorectal cancer [37,38]. The remaining studies each concentrated on a single cancer type: glioma [33], prostate [35], breast [36], lung [30], and esophageal [34] cancers.

### Observational and interventional study characteristics

We provide an overview of the observational studies (Table 3) and interventional studies (Table 4) that use mHealth technologies to evaluate CV-related measures in cancer survivors. These tables outline key aspects of each study, including the purpose, sample size, study duration, type of cancer, CV-related measures assessed, and the specific mHealth technologies used for either passive or active data collection.

**Table 3:** mHealth measures of CV health in observational studies in cancer.

Article/year Purpose	Study details	MACE/risk factors/surrogate endpoints	mHealth technologies	Passive mHealth CV measures	Active mHealth CV measures
Jonker, L.T., et al., 2021 Monitor cancer patients post-discharge utilizing a remote-home monitoring system	• **Sample size**: 58 • **Study duration**: two weeks • **Study type**: prospective cohort • **Cancer site**: mixed (solid malignant tumors; stages NS)	**Risk factors** • PA • Wt. • HR • BP **Surrogate endpoints** • Signs and symptoms indicative of CV risk (i.e., dyspnea, fever, temperature etc.) • Readmissions and post-discharge complications	• Mobile app (“*Connecare*”) which was used to monitor patient data (device-derived and self-report data) • Commercial wearable (Fitbit Charge 2; Fitbit Inc., San Francisco, CA, USA) • Other commercial devices (Nokia Withings, Issy-les-Moulineaux, France; Connecare SMS) to measure vital signs • EMR • Telephone calls were used to gather additional information regarding data collected (i.e., threshold violations)	• PA measures included daily step count and was measured using the Fitbit Charge 2 • Vital signs (e.g., HR BP and temp) were measured using monitoring devices • Readmissions and post-discharge complications were collected via the EMR	• Wt. measured via manual entry by patients into application • Symptoms measured via online questionnaires in the app regarding symptoms (i.e., dyspnea)

Abbreviations: BP, blood pressure; CV, cardiovascular; EMR, electronic medical record; GI, gastrointestinal; HR, heart rate; MACE, major adverse cardiovascular events; PA, physical activity; SaO2, oxygen saturation; temp., temperature; Wt., weight.

**Table 4:** mHealth measures of CV health in interventional studies in cancer.

Article/year Purpose	Study details	MACE/ risk factors/ surrogate endpoints	mHealth technologies	Passive mHealth CV measures	Active mHealth CV measures
Chang, E., et al., 2020. To test the feasibility of a telehealth platform to monitor CV risk factors in hematopoietic cell transplantation survivors	• **Sample size**: 18 • **Study duration**: four weeks • **Study type**: feasibility • **Cancer site**: mixed (multiple myeloma, leukemia, stages NS)	**Risk factors** • PA • Wt. • HR • BC • BG (diabetes) • BP **Surrogate endpoints** • SaO2	• BP monitor *(Model NS)* • Weight scale *(Model NS)* • Pulse oximeter *(Model NS)* • Glucometer *(Model NS)* • 4G-enabled Pod *(Model “NS”)* which transmitted data to a Telehealth Patient Portal • A commercial fitness tracker (Fitbit Charge 3; Fitbit, San Francisco, CA) • Telephone calls were utilized to update patients on alarming readings	• PA (step count) which was collected using the Fitbit • Wt. measured via Bluetooth enabled weight scale • HR and SaO2 were measured using pulse oximeter • BP measured using the BP monitor • Glucose levels were measured using the glucometer	• NA
Cheong, I.Y., et al., 2018. Evaluate the feasibility of mHealth care using a rehabilitation program for colorectal cancer patients	• **Sample size**: 102 • **Study duration**: 12 weeks • **Study type**: prospective, non-randomized • **Cancer site**: colorectal (stages NS)	**Risk factors** • PA **Surrogate endpoints** • Signs and symptoms of CV risk • HR	• Mobile app (*“Aftercare”)* supported patient self-monitoring, communication, and educational information (e.g., symptom care and exercise program) • Commercial wrist-worn fitness tracker (Partron Urban S; Partron Co, Seoul, Korea) • Survey	• PA (number of steps, walking distance) and HR during exercise were measured using the fitness tracker	• Participants completed a weekly survey in the app to report compliance to exercise program and signs and symptoms (i.e., dizziness/dyspnea)
Chow, E.J., et al., 2020. Determine the prevalence of CV disease in cancer survivors through a remotely delivered survivorship care plan	• **Sample size**: 342 • **Study duration**: 12 months • **Study type**: RCT • **Cancer site**: mixed (stages NS)	**Risk factors** • PA • Diet • Tobacco **Surrogate endpoints** • Family medical history of CV	• Telephone • Online surveys • Telehealth HIPAA-compliant web video	• NA	• Surveys were used to collect information on CV risk factors (i.e., tobacco use, PA, fruit/vegetable intake), past medical history of CV health, medication adherence, and symptoms (e.g., anxiety and depression)
Chow, E.J., et al., 2021. Determine the feasibility of a mHealth supported intervention in hematologic malignancy survivors	• **Sample size**: 41 • **Study duration**: 16 weeks • **Study type**: Pilot RCT • **Cancer site**: five years post-diagnosis of hematologic malignancy (e.g., leukemia, lymphoma, stage NS)	**Risk factors** • PA • Diet	• Mobile apps; Using one (“*Fitbit*”) to link with the fitness tracker, and the other to collect dietary data (“*Healthwatch360*”; GB HealthWatch, San Diego, California). • Commercial fitness tracker (Fitbit Flex wearable wristband; Fitbit, Inc., San Francisco, California) • Research-Grade Accelerometer (Actigraph GT3X; ActiGraph LLC, Pensacola, Florida) • Phone calls and text messages to support communication	• PA measures derived from the Fitbit included daily steps and minutes spent in MVPA • PA measures derived from the Actigraph included daily min of sedentary behavior, light-intensity PA, and MVPA	• Measures of foods and beverages consumed, and dietary components associated with CV health (i.e., sodium, saturated fats, and added sugars) were collected using the HealthWatch app
deLeuuwerk, M., et al., 2022. Evaluate the feasibility of PA self-monitoring in gastrointestinal or lung cancer patients after hospital discharge	• **Sample size**: 41 • **Study duration**: six weeks • **Study type**: feasibility study with mixed-methods design • **Cancer site**: mixed (GI and lung, stage NS)	**Risk factors** • PA	• Mobile app (*“Atris”,* Peercode BV, Geldermalsen). The app allowed patients to self-monitor PA, set goals and receive feedback • Research-grade ankle-worn accelerometer (AM400–3 axis; BV Doorwerth) • Telephone calls were used to provide motivational support and conduct interviews regarding experience with the app	• PA was measured as active minutes in 15-min bouts was collected using the accelerometer and collected data synced with app	• Users could report their activity goals through the app
Devine, K., et al., 2020^a^. To evaluate the feasibility of a technology-enhanced group fitness-based intervention among survivors of childhood cancer	• **Sample size**: 49 • **Study duration**: 12 weeks (intervention), follow up to 9 months • **Study type**: feasibility RCT • **Cancer site**: mixed (blood, brain, solid tumors, stages NS)	**Risk factors** • PA	• Mobile app (*“FitSurvivor”)* was used to provide exercise instruction, support goal setting, and participant feedback on PA • Commercial fitness tracker (Fitbit Charge or Alta) • Research-grade accelerometer (Actigraph wGT3X-BT)	• Daily min of MVPA was measured using the Actigraph accelerometer • Steps and active minutes were recorded using the Fitbit which supported self-monitoring	• PA goal setting and logging of workouts via the app
Gehring, K., et al., 2018 Investigate the feasibility of a home-based, remotely guided exercise intervention for patients with gliomas	• **Sample size**: 34 • **Study duration**: six months • **Study type**: pilot, RCT • **Cancer site**: glioma (grades II-III)	**Risk factors** • PA **Surrogate endpoint** • HR	• An online platform (*NS*) was utilized by physiotherapists to monitor patient PA and HR • Commercial fitness tracker (*Polar sports watch, Model NS*) with a HR monitor paired with platform • Telephone calls were used to assist with technical and motivational support	• PA measures included speed, distance, and HR measured via the Polar sports watch	• NA
Hassoon, A., et al., 2021 To determine whether novel AI coaching interventions increase PA among physically inactive cancer patients	• **Sample size**: 42 • **Study duration**: five weeks • **Study type**: three arm parallel RCT • **Cancer site**: mixed (lung, breast, prostate, colon, stages I-III)	**Risk factors** • PA **Surrogate endpoint** • HR	• Smart speaker (Amazon Echo, Alexa Smart Speaker) that allowed bidirectional interaction between participants and the AI virtual PA coach via voice commands (“*MyCoach”)* • Text messaging allowed unidirectional communication of the AI coach to the participant via text commands (“*SmartText*”) • Commercial fitness tracker (Fitbit Charge 2)	• PA (step count) and HR were measured by Fitbit Charge 2	• The smart speaker collected information about participants’ intention and motivation to achieve a PA goal. No data was collected in the text messaging condition.
Kadiri, S.B., et al., 2019 Test the feasibility of a home -based rehabilitation app in patients undergoing lung resection surgery	• **Sample size**: 31 • **Study duration**: 18 months • **Study design**: cohort • **Cancer site**: lung (stage NS)	**Risk factors** • PA **Surrogate endpoints** • HR • SaO2	• Tablet-based app (*“Fit 4 Surgery”*) that pulled data from the pulse oximeter and contained an exercise program • Cellular-enabled iPad mini 2 (Apple Inc. California, USA) • Bluetooth-enabled pulse oximeter (Creative PC-68B, Shenzhen Creative Industry Co. Ltd., China)	• HR and SaO2 measured via pulse oximeter	• PA data included the frequency, type and duration (minutes) of exercises completed while using the app in each session. Also, participants reported their perceived effort after completion of each exercise
Moraitis, A.M., et al., 2023 Determine the feasibility of an mHealth, home-based exercise intervention in colorectal cancer survivors	• **Sample size:** 7 • **Study duration**: 12 weeks • **Study type**: RCT • **Cancer site**: colorectal cancer (stages II-III)	**Risk factors** • PA **Surrogate endpoints** • HR	• Chest HR sensor (Polar H10) • Wrist-worn commercial fitness tracker (Polar A370) • Mobile apps. One for collecting HR data from devices (“*Polar Flow”*) and another for remote monitoring by researchers (Polar Coach app)	• PA included measures of duration and intensity of prescribed exercises, which was recorded using the Polar A370 and synchronized with the apps • HR measured using both the chest HR sensor (more accurate) and wrist-worn fitness tracker	• Participants reported the type of PA (i.e., weight-bearing exercise) chosen, but it was unclear how this data was captured^b^
Papadopoulos, E., et al., 2022 Assess the effects of different exercise delivery modes in prostate cancer survivors	• **Sample size**: 37 • **Study duration**: six months • **Study type**: RCT • **Cancer site**: prostate (all stages)	**Risk factors** • PA **Surrogate endpoints** • HR • Signs and symptoms of CV risk	• Smartphone • Mobile app (“*Connected Wellness Platform*”, NexJ Systems Inc., Toronto, ON). The app was used to collect data and to communicate with health coach • Commercial HR monitor (Polar, NY, USA) • Research-Grade Accelerometer (Actigraph GT3X, Pensacola, FL)	• PA was measured using an Actigraph, data was extracted in 60-s epochs, and outcome was time spent in MVPA • HR measured using a Polar HR monitor	• The Connected Wellness Platform enabled participants to input health information, log symptoms, and track their exercise routines
Uhm, K.E., et al., 2017 Investigate the effects of mHealth compared to a conventional exercise program in breast cancer patients	• **Sample size**: 356 • **Study duration**: 12 weeks • **Study type**: prospective, multicenter quasi-RCT • **Cancer site**: breast (all stages)	**Risk factors** • PA	• Mobile app (“*Smart After Care”;* BIT Computer Co., Ltd., Seoul, Korea) used to collect data on weekly PA progress and goals, and to educate about the prescribed exercise regimen • Commercial fitness tracker (*InBodyBand* by InBody Co., Ltd., Seoul, Korea)	• Minutes of engaging in PA was measured using the InBodyBand pedometer. Resulting data was imported into the smartphone app	• Participants entered into the app the number of sets for each resistance exercise performed
Yang, K., et al., 2021 Test the feasibility of a health coaching mobile application in esophageal cancer patients	• **Sample size**: 36 • **Study duration**: eight weeks • **Study type**: prospective single-arm pilot • **Cancer site**: esophageal (stage NS)	**Risk factors** • PA • Wt. • Diet	• Health coaching mobile app *(“Noom”)* which provided advice on health behaviors and allowed participants to record daily diet, PA (i.e., exercise), and Wt. data	• PA (i.e., step count) was passively measured using the smartphone	• Diet, Wt., and PA measured via manual entry into app by users

Abbreviations: AI, artificial intelligence; BC, body composition; BP, blood pressure; BG, blood glucose; CV, cardiovascular; GI, gastrointestinal; HR, heart rate; MACE, major adverse cardiovascular events; MVPA, moderate to vigorous physical activity; NA, not applicable; NS, not specified; PA, physical activity; RCT, randomized control trial; SaO2, oxygen saturation; Wt., weight.

Superscripts: ^a^, this study contained children and adults; ^b,^ included although it was unclear how the data was captured.

### Quality assessment

**Fig 4:**
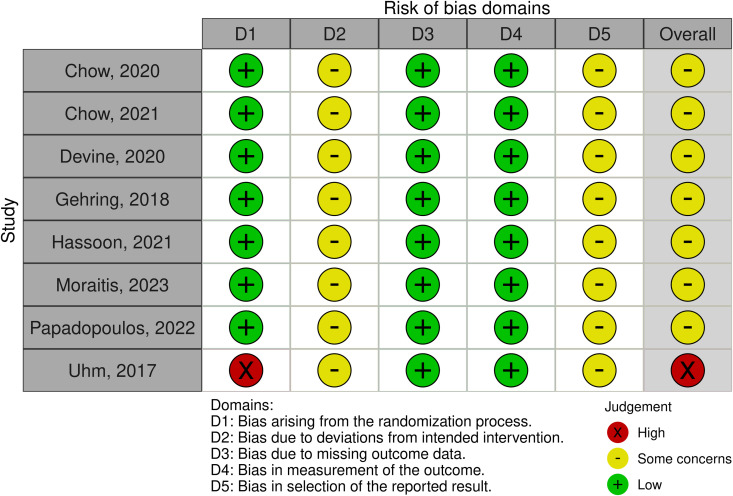
Randomized control trials were appraised using the Revised Cochrane risk-of-bias (RoB-2).

**Fig 5:**
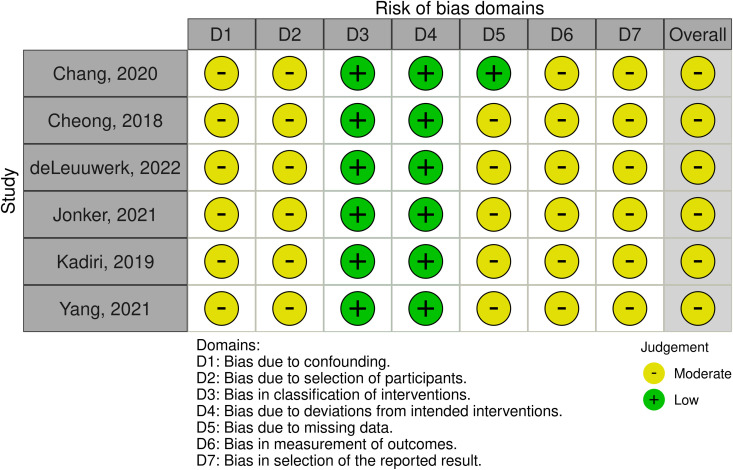
Observational and non-randomized studies were appraised using the ROBINS-I tool.

Of the eight randomized controlled trials (RCTs) included in this review, seven studies were assessed as having some concerns of bias, and one study was identified as being at a high risk of bias, according to RoB-2 (Fig 4). Among the six non-randomized studies, all were determined to be at moderate risk of bias, including the observational study, as assessed by the ROBINS-I (Fig 5). These findings indicate that while many studies demonstrated reasonable methodological quality, a notable proportion carried a potential for bias, which necessitates cautious interpretation of their results. Specifically, studies with ‘some concerns’ or ‘high risk’ of bias (RoB-2) and ‘moderate risk’ (ROBINS-I) may present findings where the true effect of the mHealth intervention could be either overestimated or underestimated. For instance, concerns regarding the randomization process, deviations from intended interventions, or unblinded outcome assessment in RCTs could inflate reported intervention effects. Similarly, in non-randomized studies, issues such as confounding, selection bias, or deviations from intended interventions could significantly impact the internal validity of the observed associations between mHealth use and CV outcomes. Therefore, while these studies contribute to the overall evidence base, the presence of these biases reduces the certainty in their reported outcomes and limits the extent to which their findings can be generalized without reservation.

A significant source of bias across all studies was the lack of blinding for outcome assessors, interventionists, and the participants undergoing the intervention. Given the nature of the interventions (primarily exercise-based programs, mHealth applications, and activity monitoring technologies), blinding was inherently not feasible, leading to potential performance bias. Another frequent source of bias was deviations from intended interventions, as adherence to interventions varied across studies. Several studies relied on self-directed home-based exercise programs or wearable activity monitors, where participants’ engagement with the intervention was not strictly controlled. Furthermore, selective reporting was a concern across all studies, as many relied on patient-reported outcomes (PROs) and mHealth technology to measure and track CV-related data. This introduced potential bias, as participants had access to their own data, which may have influenced their behavior, engagement, and accuracy of self-reporting. Moreover, because most of these studies aimed to evaluate the feasibility and acceptability of the interventions rather than exploring causal relationships, the risk of bias from confounding variables was also notable. Overall, though the majority of the studies were at moderate risk of bias, these concerns were largely due to the nature of behavioral interventions, reliance on self-report measures, and the difficulty of blinding participants and providers rather than fundamental flaws in study design.

### Types of CV measures collected using mHealth

**Fig 6:**
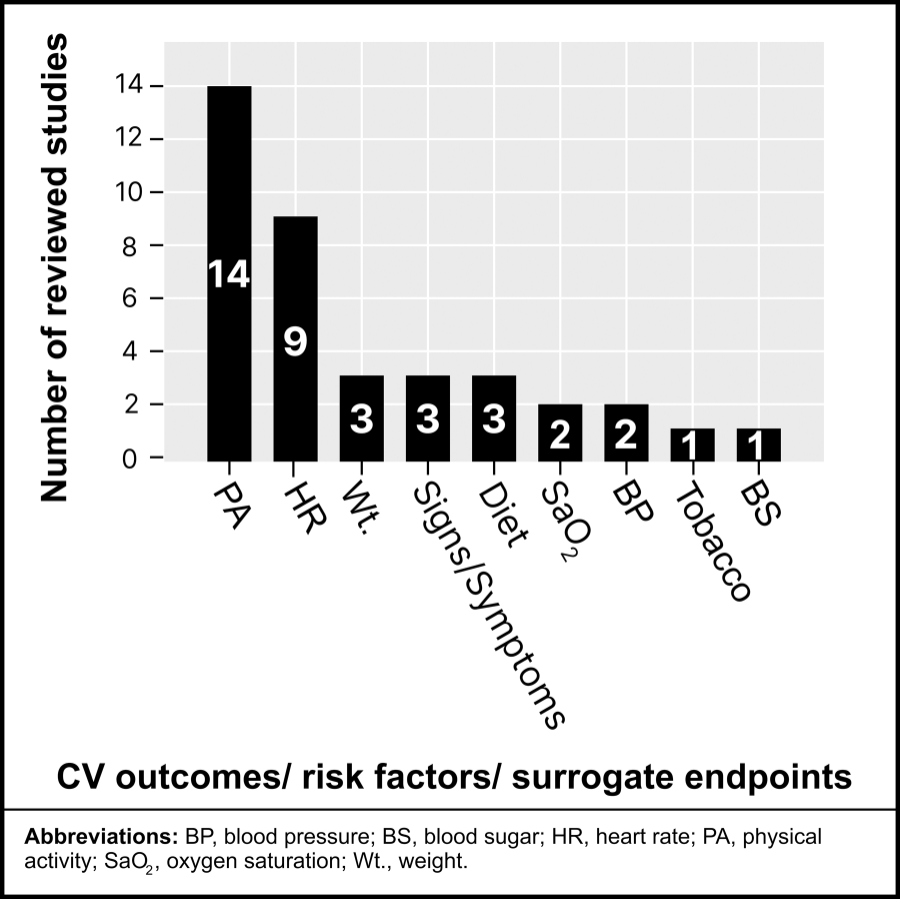
Measures of CV risk factors using mHealth technology.

Fig 6 below summarizes CV outcomes, risk factors, and surrogate endpoints measured in the reviewed studies. PA was identified as the most common CV risk factor assessed across all reviewed studies [23,28–40], followed by HR monitoring as the second most common measure [23,28,30,33,35,37–40]. Other CV-related measures were less frequently reported. Weight [23,34,40], signs/symptoms [35,38,40], and dietary factors [29,31,34] were moderately represented, while traditional clinical markers such as oxygen saturation (SaO_2_) [23,30], BP [23,40], tobacco use [29], and blood glucose [23] were underutilized in comparison (*see* Fig 6 for detailed distribution.

### Prevalence of types of mHealth technologies

**Fig 7:**
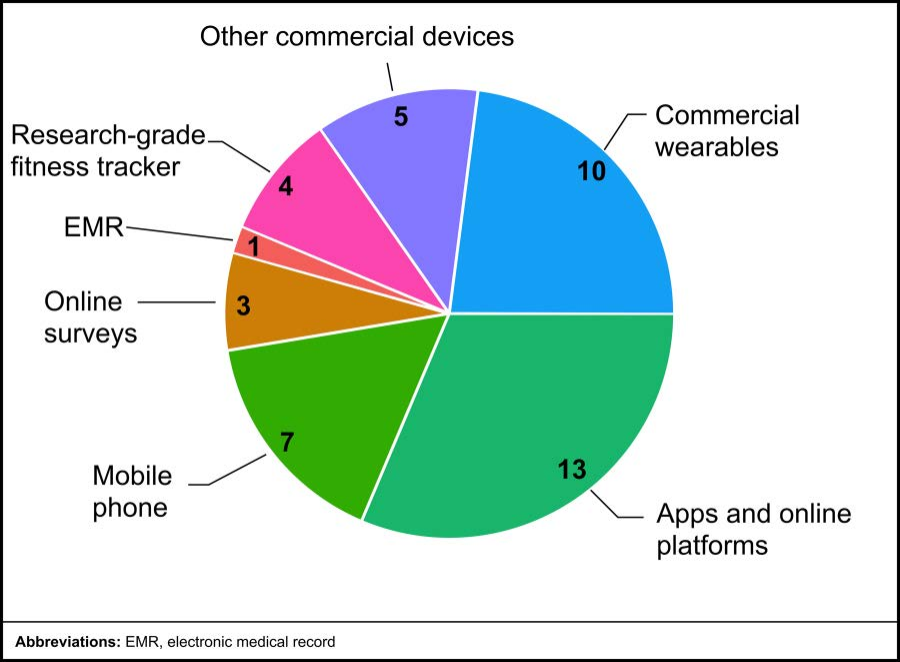
mHealth technology distribution among reviewed studies.

The studies included in this review employed a range of mHealth technologies to assess CV health in cancer survivors, with the distribution of technologies summarized below (see Fig 7). The most commonly used mHealth technology was apps and online platforms, featured in 13 studies that demonstrated their central role in facilitating health monitoring, symptom tracking, and behavioral interventions [23,29–40]. Mobile apps [30–32,34–40] and platforms [23,29,33] were used for active (e.g., symptom reporting) and passive data collection (e.g., integration with mobile devices). Commercial wearables, such as fitness trackers and smartwatches, were utilized in 10 studies, highlighting their widespread adoption as tools for tracking PA and other health-related metrics [23,28,31,33,35–40]. Other commercial devices, including Bluetooth-enabled BP cuffs, smart speakers, and digital weight scales, were employed in five studies [23,28,30,35,40]. These devices typically collect passive data, supporting their integration into mHealth ecosystems. Research-grade fitness trackers (also called activity trackers), such as ActiGraph and activPAL, were used in four studies, reflecting their value in providing precise, validated measures of activity and sedentary behavior for research purposes [31,32,35,39]. Electronic medical records were included in only one study, suggesting limited integration of EMR data in the context of mHealth for CV-health assessment [40]. Three studies utilized online surveys, primarily when integrated with mobile platforms or apps to collect active participant data [29,38,40]. Lastly, mobile phones, used to support telephone calls and text messaging for remote monitoring or counseling, were employed in seven studies [23,28,29,31–33,40].

### Types of passive data collected via mHealth

**Fig 8:**
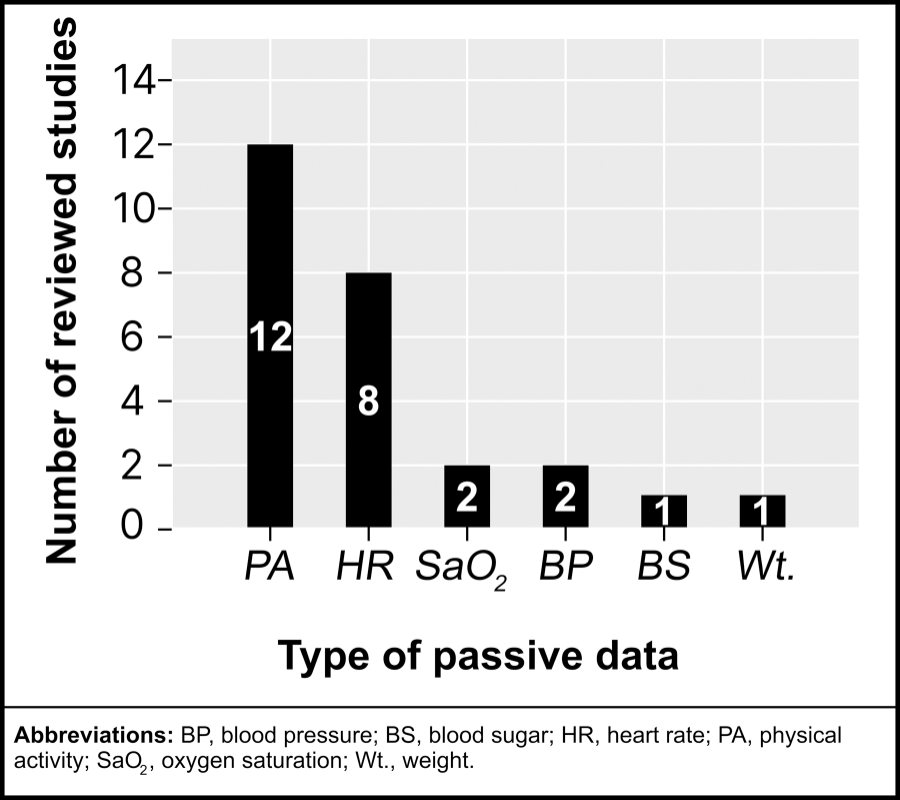
Reviewed studies categorized by passive mHealth data.

The reviewed studies employed passive mHealth technologies to gather various CV measures (see Fig 8), showcasing the automated nature of data collection with minimal user input. The most frequently collected type of passive data type was PA, recorded in 12 studies [19–23,25–31], underscoring its importance as a vital CV function or health metric. Passive PA data typically encompassed measures such as step count, walking distance, speed, and time spent at different intensity levels (minutes in sedentary, light-intensity, or moderate to vigorous PA).

HR was the second most frequently monitored parameter [23,28,30,33,35,37,38,40], passively collected in eight studies. This was followed by SaO_2_ [23,30] and BP [23,40], each recorded in two studies. Less commonly assessed measures included blood glucose [23] and weight [23], each recorded in a single study via Bluetooth-enabled devices. Notably, no studies collected data on body temperature passively.

### Types of active data collected via mHealth

**Fig 9:**
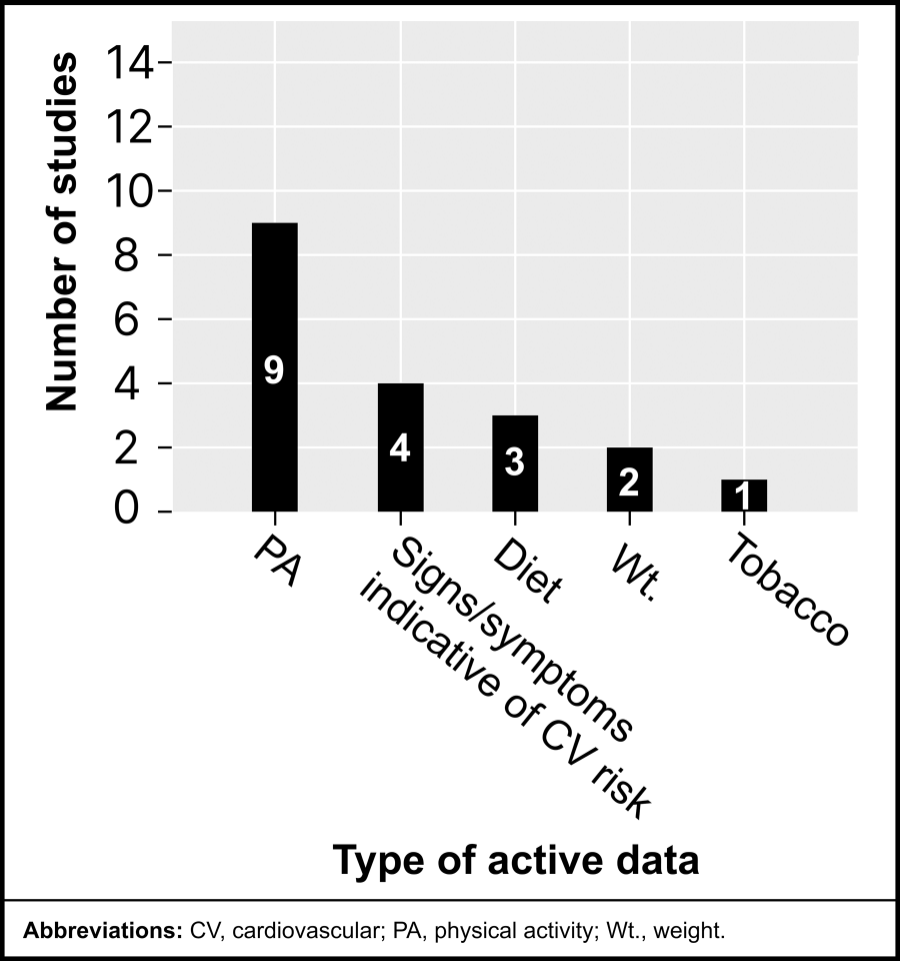
Reviewed studies categorized by active mHealth data.

The reviewed studies also utilized active mHealth technologies to collect CV measures (*see* Fig 9), requiring participants to input or report information manually. The most frequently collected active data was PA, recorded in 9 studies [28–30,32,34–37,39], highlighting its significance as a participant-reported measure for CV health assessment. The types of active PA data collected typically included setting and tracking of goals, logging types and routines of PA, and conducting surveys about exercise program compliance.

Signs and symptoms were the second most frequently reported data type, actively collected in four studies [29,35,38,40], often as part of symptom tracking or self-monitoring interventions. Diet was reported in three studies [29,31,34], reflecting its relevance to lifestyle and CV health management.

Weight was actively collected in two studies [34,40], typically through participant entry into apps or digital platforms. Tobacco use was documented in one study [29]. Notably, no studies actively collected data on HR, blood SaO_2_, BP, or temperature, suggesting these metrics were primarily recorded passively when included.

These findings underscore the reliance on participant-reported lifestyle and symptom data for active mHealth data collection, complementing the passively collected physiological metrics.

## Discussion

This is the first systematic review to comprehensively characterize how mHealth technologies are used to monitor a broad range of CV health indicators and risk factors, specifically in cancer survivors. While previous reviews have examined the use of mHealth for promoting PA and improving cardiorespiratory fitness in cancer survivors [41] or the general use of wearables in oncology [10], our review provides a detailed synthesis of the specific CV metrics being tracked, the types of technologies employed, and the distinction between active and passive data collection methods. Our findings show a strong emphasis on monitoring PA and HR, primarily through mobile apps and commercial wearables, yet reveal significant gaps in the tracking of other key markers like BP, sleep, and glycemic variability. This review thus establishes a critical baseline that highlights both progress and untapped opportunities for leveraging mHealth to improve CV outcomes in cancer populations. This review also adds to the emerging research exploring how, by leveraging mHealth technologies [9,11,12,41], healthcare providers and interventionists can monitor key health metrics, promote evidence-based interventions, and address shared risk factors between CVD, cancer, and cancer treatments.

Apps and online platforms were the most widely used mHealth technologies, facilitating active and passive data collection, followed by commercial wearables and other commercial devices like Bluetooth-enabled devices. Despite the versatility of mHealth, certain metrics such as body temperature and other physiological markers were underutilized, highlighting opportunities for future research. It’s important to note that the underutilization may be due not only to the availability of the API derived from the device but also to researchers’ ability to extract and use the data. Thus, our group has recently published a manuscript that includes recommendations, considerations, and code to extract data from commercial activity monitors in cancer [42].

Passive methods were more frequently employed in collecting CV-related health data, particularly for PA, HR, BP, and SaO2, reflecting the efficiency of wearable and connected devices for near real-time, low-burden monitoring. In contrast, active data collection was mainly used for PA (including behaviors that could not be captured passively), signs, symptoms, and diet due to the need for participant-reported context. While passive methods provide continuous, objective tracking, integrating active self-report measures remains essential for capturing qualitative health behaviors (e.g., dietary habits, resistance training), that would otherwise be challenging or impossible to obtain passively. The advantages of active and passive approaches highlight their utility in mHealth applications, particularly for tracking CV health in a growing patient population at risk for adverse CV outcomes.

### mHealth-based CV risk monitoring

mHealth technologies provide unique opportunities for the early detection and management of CV risk factors, which is critical for cancer survivors undergoing cardiotoxic therapies [43,44]. For example, photoplethysmography (PPG) sensors in commercial smartwatches and activity monitors now feed their software algorithms, which are capable of detecting irregularities in heart rhythm, enabling the early identification of cardiac arrhythmias and conditions such as atrial fibrillation, which are common cardiotoxic side effects during and after cancer treatment [45]. Beyond early MACE detection, passive data from wearables can reveal subtle changes in PA, mobility patterns (i.e., daily distance traveled) and sleep quality, which have been shown to serve as ‘digital biomarkers’ that can provide early warnings of subclinical cardiotoxicity that may manifest as increased fatigue or reduced functional capacity before overt symptoms appear [46–48]. Gregory et al. conducted a qualitative study and identified themes that highlighted the potential of symptom-tracking apps to enable real-time reporting of adverse effects such as fatigue, palpitations, or dyspnea, facilitating timely clinical interventions [9]. Wearable devices that passively monitor HR, SaO_2_, and PA can enhance active data collection by providing continuous objective insights into a survivor’s health status. As reported throughout this review, CV health can be assessed using direct metrics (e.g., HRV, SaO_2_, BP) and indirect indicators (e.g., adverse patterns in PA, sleep disturbances, or symptom fluctuations).

Several key physiological markers remain underutilized in mHealth-based CV monitoring [49]. These markers, if integrated into mHealth monitoring systems, could offer additional insights into CV risk profiles, particularly in populations with underlying metabolic disturbances, cancer treatment-related toxicities, or autonomic dysfunction. For example, HRV has been extensively studied in CV medicine and is recognized as an independent predictor of cardiac events [50,51], yet more research is needed to understand its application in cancer. Moreover, sleep disturbances and dysregulated stress responses are common among cancer survivors [52,53], and can contribute to CV dysfunction. Evidence suggests that poor sleep is associated with higher CV risk [54], which also may compound the cardiotoxic effects of cancer treatments. Similarly, glycemic variability has been increasingly recognized as a stronger predictor of CV events than traditional fasting glucose levels [55], indicating the need for continuous rather than single-point blood glucose monitoring. Integrating these markers into wearable-enabled, AI-driven CV monitoring systems could potentially enhance early risk detection and targeted intervention strategies.

Beyond technological advancements, behavior-based strategies are equally critical for improving CV health in cancer survivors. Physical activity plays a vital role in mitigating treatment-related CV risk, aligning with the “Exercise is Medicine” initiative [56,57]. This approach emphasizes exercise as a fundamental component of cancer recovery and CV disease prevention, reinforcing the need for mHealth-driven PA interventions tailored to cancer survivors.

### Exercise is medicine: the role in cancer and CV health

Physical activity and exercise are key behavioral targets for promoting positive health outcomes in cancer and CV health, which aligns with the “Exercise is Medicine” initiative [56,57]. Exercise can be used to manage cancer-related side effects and plays a pivotal role in reducing CV risk and improving survivorship outcomes [57]. The American College of Sports Medicine (ACSM) has published a series of complementary reviews [57–59], one of which includes specific evidence-based exercise prescriptions or recommendations to manage common cancer symptoms, including but not limited to anxiety, depression, fatigue, quality of life, and sleep impairments [58].

Our systematic review revealed that PA was the most monitored measure of CV health, reflecting its importance as a modifiable risk factor. mHealth tools, such as wearable devices and mobile apps, provide scalable methods for tracking PA and engaging survivors in tailored supportive care interventions [60]. With the continuous rise of emerging technologies alongside advanced computational predictive models, there is a unique opportunity to create tailored and automated (when appropriate) PA interventions to promote CV health among cancer survivors. Future mHealth interventions should align with exercise guidelines for cancer survivors to provide personalized recommendations that account for personal limitations and preferences while optimizing CV-related health.

### Importance of life’s essential 8 health markers in cancer

The findings of this review align with the AHA framework of Life’s Essential 8, centered on the modifiable determinants of CV health (i.e., PA, diet, BP, blood glucose, cholesterol, weight, sleep, and nicotine avoidance) for optimizing CV health [6,7]; high adherence to these metrics has been shown to significantly reduce the risks of all-cause, cancer-specific, and non-cancer mortality in cancer survivors [61]. Furthermore, maintaining optimal CV health based on LE8 can mitigate shared risk factors between CVD and cancer, such as obesity, hypertension, and poor diet, emphasizing the interconnected nature of these conditions [61]. Koene et al. further emphasized that these shared risk factors point to biological and behavioral overlaps between CV disease and cancer, driven by chronic inflammation, oxidative stress, and other pathways [8]. This underscores the need for integrated prevention strategies that target both diseases simultaneously.

The SPHERE study (Stroke Prevention in Healthcare Delivery Environments) assessed the integration of an EHR-based cardiovascular health (CVH) assessment tool in primary care settings to improve CV risk factor discussions between providers and patients [62,63]. The study focused on Life’s Simple 7 metrics (i.e., smoking, BMI, PA, diet, cholesterol, BP, and blood glucose), and the application generated visualizations of the CVH score within the EHR during the patient-provider encounters. The study evaluated changes in these metrics over one year among older female patients. Results showed modest improvements in BMI and diabetes control, but no significant changes in smoking, total cholesterol, or BP, highlighting the need for more comprehensive interventions that utilize patient’s everchanging data. This study is relevant as it illustrates early efforts to integrate digital tools for CV risk assessment, and it underscores the need for mHealth and AI-driven solutions beyond static EHR assessments.

The SMART-BREAST trial was one article that did not meet the eligibility criteria but was highly relevant and worth mentioning [11,12]. This study demonstrated the potential of smartphone-based interventions to enhance CV outcomes among breast cancer survivors through step-tracking and CV risk reduction strategies. Over 12 months, participants significantly improved their 6-minute walk test distance, highlighting how structured mHealth interventions can drive meaningful health improvements. Integrating personalized, real-time feedback systems into survivorship care could facilitate the systematic monitoring of LE8 metrics, supporting both CV health and overall well-being in this vulnerable population.

### Integrating mHealth with EMRs and clinical workflows

One of the most persistent challenges in scaling mHealth technologies is integrating them meaningfully into clinical workflows. Without thoughtful alignment, continuous or passive data from mobile apps and wearables can overwhelm providers with data and go unused.

Several examples now illustrate pathways toward practical integration. For instance, My Wellness Check, a digital symptom-monitoring platform embedded within the electronic medical record (EMR) at a major U.S. cancer center, enables patients to report key outcomes such as distress, physical function, and unmet needs in advance of their visit [64]. Care teams then receive alerts and tailored action plans to guide follow-up. This system has helped operationalize patient-centered care and facilitate team-based responses to mHealth inputs [65].

Another example is the implementation of Life’s Simple 7, an evidence-based CV health tool, within oncology settings. When combined with EMR-linked platforms, it supports structured conversations around risk factor modification for cancer patients at increased CV risk [65].

Furthermore, results from a recent study by Natori et al. [64] found that an EMR-integrated digital monitoring tool used across multiple ambulatory oncology clinics improved patient-provider communication and was associated with downstream improvements in care delivery, including reductions in emergency department visits, hospitalizations, and length of stay. These findings reinforce the clinical value of EMR integration and the importance of workflow-aligned mHealth strategies in supporting scalable survivorship care.

Technology-based interventions can increase access to care and improve symptom management, particularly for survivors in rural areas or those with limited mobility. However, a paradox exists where the challenges patients face may reduce their capacity to engage with these helpful tools. While digital health monitoring is often feasible, certain patient characteristics are associated with lower engagement. For instance, in a study of a diverse sample of ambulatory oncology patients, those who were older, male, Hispanic/Latino, or living without a partner were less likely to complete digital questionnaires [64]. Older patients, who may have an increased prevalence of comorbidities and mobility issues, can find it more difficult to cope with their unmet needs [66]. This highlights a critical challenge for intervention design: the need to adapt digital health tools to overcome barriers and meet the needs of all patients, especially those who are older or managing complex health issues.

### Quality and risk of bias of reviewed studies

While our findings highlight the potential of mHealth technologies, it is crucial to interpret the evidence within the context of the methodological quality of the included studies. The risk of bias assessment revealed that a substantial number of studies were identified as having ‘some concerns,’ ‘moderate,’ or ‘high’ risk of bias. These findings underscore the need for cautious interpretation of the reported intervention effects. Specifically, issues such as inadequate blinding, concerns regarding the randomization process, deviations from intended interventions, and potential for confounding in non-randomized studies could lead to misrepresentation of the true efficacy of mHealth interventions.

The challenges in achieving full blinding in mHealth interventions are often inherent due to their nature. Unlike pharmacological trials, where a placebo can be physically indistinguishable from the active treatment, mHealth interventions typically involve active engagement with a digital tool (e.g., a mobile app, wearable device). Participants are generally aware they are using a specific technology or receiving an interactive program, making it challenging to blind them to their allocation status. Similarly, those delivering the intervention (e.g., coaches, health educators interacting via the app) or assessing direct user engagement data cannot be fully blinded to the intervention arm. This is particularly true for interventions that require participants to actively log data or receive personalized feedback, as the intervention itself is often the “treatment”.

When participants are aware of receiving an mHealth intervention, their behavior might be influenced, potentially leading to a placebo effect that artificially inflates perceived benefits (performance bias). Similarly, unblinded intervention deliverers or outcome assessors might inadvertently influence participant behavior or data collection/analysis in a way that aligns with the study’s hypotheses. This heterogeneity in methodological quality limits the overall certainty of the evidence and the generalizability of findings across diverse populations and settings.

Moving forward, future research in mHealth for CV health should prioritize rigorous methodological designs to enhance the quality and reliability of evidence. Specifically, we recommend: 1) employing appropriate blinding strategies for participants, personnel, and outcome assessors whenever feasible, acknowledging the inherent challenges in mHealth interventions and developing innovative solutions to mitigate unblinding effects, 2) conducting larger, multi-site studies with longer follow-up periods to better assess the sustained impact and generalizability of mHealth interventions on CV outcomes, 3) utilizing appropriate statistical methods to account for confounding in non-randomized studies and to handle missing data effectively, and 4) increasing the use of objective, passively collected data where appropriate (e.g., wearable sensor data for PA, HR) to complement self-reported measures and reduce recall or social desirability bias.

### Gaps and future directions in cardio-oncology monitoring

Future research should explore the integration of these underutilized measures into health monitoring systems to provide a more comprehensive assessment of cardiovascular risk in cancer survivors.

Expanding the use of real-world monitoring (e.g., continuous tracking of HRV, sleep, glycemic variability) could provide a deeper understanding of how CV health fluctuates in response to cancer treatments and environments (e.g., physical, social, etc.). AI-driven analytics offer an opportunity to leverage multi-sensor data streams for personalized risk trajectory modeling, enabling earlier interventions and more effective CV risk management. For example, given our finding that PA and HR were frequently captured metrics by mHealth technologies, clinicians can implement specific AI-driven decision support systems that automatically flag concerning patterns, triggering structured care protocols, including telehealth appointments or cardio-oncology consultations. mHealth technologies have been shown to be capable of revealing subtle changes in ‘digital biomarkers’ (based on PA or HR patterns) that can provide early warnings of subclinical cardiotoxicity [46,47], and clinicians may be able to utilize machine learning based notification systems that further facilitate alterations of the clinical treatment plan (such as dose modifications, echocardiogram scheduling, or ACE inhibitor initiation) to pre-emptively prevent future MACEs.

Further, the heterogeneity in cancer types, study designs, and reporting methods across studies poses challenges in drawing generalizable conclusions. Future research should prioritize the use of standardized CV risk assessment tools and outcome measures to enhance comparability. Incorporating subgroup analyses and conducting multi-center RCTs may further improve the specificity and generalizability of findings in this field.

Additionally, disparities in digital health access, including financial and geographical barriers, as well as digital literacy gaps, must be addressed to ensure the equitable implementation of mHealth interventions across diverse patient populations. Developing user-friendly, low-burden wearable solutions for tracking could improve adherence and expand the feasibility of integrating these measures into routine clinical monitoring.

### Data privacy and security challenges in mHealth

Adoption of mHealth technologies in clinical practice must also address critical concerns related to data privacy, security, and regulatory compliance. These tools frequently collect sensitive personal health data, often including biometric or geolocation information, which raises significant questions about data storage, sharing, and protection. The European Union enforces stringent standards under the General Data Protection Regulation (GDPR), which imposes clear requirements for obtaining user consent, minimizing data collection, and ensuring the secure transfer of data across borders. In contrast, the United States lacks a unified federal data protection framework. While the Health Insurance Portability and Accountability Act (HIPAA) governs healthcare data within covered entities, it does not apply broadly across all mHealth platforms. Only a few states, most notably California through the California Consumer Privacy Act (CCPA) and California Privacy Rights Act (CPRA), have enacted comprehensive privacy legislation. This fragmented approach contributes to vulnerabilities in real-world implementation and limits cross-border research collaborations.

The lack of alignment between GDPR and U.S. federal policies has created well-documented barriers to international data sharing, particularly with public institutions like the NIH, which are not subject to EU-style data subject rights or legal remedies due to sovereign immunity. Even recent efforts, such as the EU-U.S. Data Privacy Framework, face legal uncertainty and criticism for insufficient safeguards. These challenges underscore the need for mHealth developers, researchers, and policymakers to collaborate on security protocols, user-centered consent mechanisms, and interoperable data governance frameworks that can meet evolving global standards [67–69].

## Limitations

While this review highlights the potential of mHealth technologies in monitoring CV health among cancer survivors, some limitations should be noted. The heterogeneity in cancer types, study designs, and reporting methods makes direct comparisons across studies challenging. This heterogeneity prevents conducting a formal meta-analysis and assessing the comparative effectiveness of different mHealth technologies or intervention strategies. As a result, these findings are necessarily limited to a descriptive characterization of the current landscape of mHealth applications. To overcome this limitation in the future, studies should aim to focus on particular populations to understand the acceptability and feasibility of using this technological approach. Also, researchers should implement standardized and validated instruments (e.g., CV risk assessment tools such as the Framingham risk score and the risk for atherosclerotic cardiovascular disease (ASCVD Risk Estimator Plus) to ensure consistent and comparable outcome reporting [70,71]. In addition, a greater emphasis on funding larger, multi-center RCTs with standardized protocols and pre-specified subgroup analyses based on cancer type, treatment history, and comorbidity burden is needed to generate more robust, generalizable evidence.

Also, our focus on predefined aspects of interest may have resulted in less emphasis on certain technical details, such as specific mHealth device specifications and data processing methods. Our search strategy focused on the terms “mHealth” or “mobile health.” We recognize that some studies may use more general terms such as “app” or “mobile application,” and broadening our search to include these search terms could have captured additional relevant studies. Future reviews may consider expanding the search strategy to capture these variations, although this may require additional filtering to maintain methodological relevance. Additionally, our eligibility criteria excluded some relevant articles (e.g., protocols, reviews, letters) that discuss digital health applications in CV monitoring. This decision, while necessary to maintain the methodological rigor of a targeted systematic review, means our synthesis may not fully capture the breadth of innovation in the field. However, insights from these sources still inform the broader field. For instance, Murphy et al. published both a protocol [12] and findings (published as a letter) [11] on a smartphone-based model of care or intervention aimed at promoting exercise, reducing CV risk, and fostering community engagement among women undergoing breast cancer treatment. By excluding such articles, our review may underrepresent the impact of promising interventions that have been reported in preliminary or non-traditional formats. Similarly, study protocols often contain rich methodological details that are not present in the final publications, and narrative reviews provide essential context on emerging trends. Future knowledge synthesis projects, such as scoping or narrative reviews, could be designed specifically to analyze these varied sources, offering a broader perspective that complements the focused findings of systematic reviews.

Additionally, a meta-analysis was not conducted, as the primary aim of this review was to characterize the use of mHealth technologies rather than quantify their clinical impact. This review was not designed to compare the effectiveness of different mHealth technologies (e.g., apps, wearables, Bluetooth-enabled devices). Given the wide heterogeneity in study aims, populations, intervention components, and outcomes assessed, such comparisons would not only be methodologically inappropriate but could also mislead readers. Comparative effectiveness requires standardized designs and head-to-head evaluations, which were not present in the included studies. As the field evolves and more rigorously controlled trials accumulate, future reviews may be better positioned to examine relative effectiveness across mHealth tools*.*

Finally, most studies did not report on the presence or impact of comorbidities, which may limit generalizability to survivors with multimorbidity. However, evidence from digital interventions in survivors with advanced prostate cancer, a population with a high comorbidity burden, suggests that such individuals can meaningfully engage in technology-based support and experience improvements in symptoms. Prior work has also noted that while poor health status can sometimes hinder digital engagement, for many, it may also increase the need for remote care solutions [72–74].

## Conclusion

This review reveals that current mHealth applications in cardio-oncology research predominantly leverage mobile apps and commercial wearables to monitor PA and HR. This characterization of the landscape provides a foundational understanding of how these tools are being deployed, highlighting the value of passive data collection for continuous, low-burden tracking.

These findings have significant implications for clinical practice, demonstrating that while feasible tools exist for remote monitoring, their full potential is unrealized due to challenges in EMR integration and a narrow focus on a few key metrics. Future research should therefore prioritize the integration of underutilized but clinically valuable markers, such as HRV and sleep patterns, and focus on standardizing data collection to improve comparability across studies. By building on current applications to address these specific gaps, mHealth technologies can significantly enhance the precision of CV health monitoring, supporting more timely and personalized survivorship care. However, realizing this potential is contingent on overcoming the significant implementation barriers identified in this review, including EMR integration and the need for broader data standardization.

## Supporting information

S1 Checklist PRISMA Checklist. (DOCX)
